# Differentiation in the protein synthesis-dependency of persistent synaptic plasticity in mossy fiber and associational/commissural CA3 synapses *in vivo*

**DOI:** 10.3389/fnint.2013.00010

**Published:** 2013-03-01

**Authors:** Hardy Hagena, Denise Manahan-Vaughan

**Affiliations:** ^1^Department of Neurophysiology, Medical Faculty, Ruhr University BochumBochum, Germany; ^2^International Graduate School for Neuroscience, Ruhr University BochumBochum, Germany

**Keywords:** associational-commissural fibers, CA3, mossy fibers, protein-synthesis, transcription

## Abstract

Long-term potentiation (LTP) and long-term depression (LTD) are two mechanisms involved in the long-term storage of information in hippocampal synapses. In the hippocampal CA1 region, the late phases of LTP and LTD are protein-synthesis dependent. In the dentate gyrus, late-LTP but not LTD requires protein synthesis. The protein synthesis-dependency of persistent plasticity at CA3 synapses has not yet been characterized. Here, the roles of protein transcription and translation at mossy fiber (mf) and associational/commissural (AC)- synapses were studied in freely behaving rats. In control animals, low-frequency stimulation (LFS) evoked robust LTD (>24 h), whereas high-frequency stimulation (HFS) elicited robust LTP (>24 h) at both mf-CA3 and AC-CA3 synapses. Translation inhibitors prevented early and late phases of LTP and LTD at mf-CA3 synapses. In contrast, at AC–CA3 synapses, translation inhibitors prevented intermediate/late-LTP and late-LTD only. Transcription effects were also synapse-specific: whereas transcription inhibitors inhibited late-LTP and late-LTD (>3 h) at mf-CA3 synapses, at AC–CA3 synapses, protein transcription affected early-LTP and late-LTD. These results show that the AC-CA3 and mf-CA3 synapses display different properties in terms of their protein synthesis dependency, suggesting different roles in the processing of short- and long term synaptic plasticity.

## Introduction

Many of the synaptic modifications that support persistent changes in synaptic strength require *de novo* protein synthesis. Protein synthesis, in turn, underlies many forms of long-term memory (Davis and Squire, 1984; Abraham and Williams, 2003; Sutton and Schuman, 2006). In the hippocampus, one of the most important structures for declarative memory formation, functional differentiation has been proposed for its neuroanatomically-defined subregions. Whereas the dentate gyrus is believed to engage in pattern separation (Treves and Rolls, 1992; O'Reilly and McClelland, 1994; Gilbert et al., 2001), the CA3 region may enable pattern completion (Marr, 1971; Nakazawa, 2002). CA1 may mediate error detection (Vinogradova, 2001; Lisman and Grace, 2005; Kumaran and Maguire, 2007) and the generation of an integrated spatial representation (Goodrich-Hunsaker et al., 2008). The main mechanisms underlying persistent synaptic information storage, and therefore perhaps memory, comprise long-term potentiation (LTP) and long-term depression (LTD). These forms of synaptic plasticity display different dependencies on protein translation and transcription, depending on the hippocampal subregion investigated (Krug et al., 1984; Frey et al., 1988; Huang et al., 1994; Nguyen et al., 1994). This may reflect functional differentiation of the roles LTP and LTD play in the generation of memory engrams. Indeed, it has been reported that expression of persistent LTP is associated with acquisition of knowledge about space, whereas LTD is associated with learning about spatial context (Kemp and Manahan-Vaughan, 2007, 2008; Hagena and Manahan-Vaughan, 2011).

The role of protein synthesis in these forms of long-lasting plasticity in the CA3 region of intact animals has not yet been explored. Whether persistent synaptic plasticity in CA3 depends on protein synthesis is an important question as the CA3 region is believed to play a unique role in memory formation. Neuroanatomically, the CA3 pyramidal cells receive input from mossy fibers that terminate on the proximal portion of dendrites (Blackstad and Kjaerheim, 1961; Amaral, 1979) and express an N-methyl-D-aspartate receptor (NMDAR)-independent form of LTP (Harris and Cotman, 1986; Zalutsky and Nicoll, 1990). Expression of this form of LTP depends on presynaptic mechanisms (Staubli et al., 1990; Xiang et al., 1994; Weisskopf and Nicoll, 1995). Furthermore, LTD that is elicited by low-frequency stimulation (LFS), is preceded by potent facilitation of synaptic responses (called frequency facilitation) that is not seen at other hippocampal synapses (Salin et al., 1996; Toth et al., 2000; Moore et al., 2003; Hagena and Manahan-Vaughan, 2010). The role of mossy fiber (mf) plasticity in memory is unknown-however, the unique properties of frequency facilitation suggest it may play a role in working memory and/or informational integration. CA3 pyramidal cells also receive input from associational fibers originating from CA3 cells of the ipsilateral hemisphere and from commissural fibers of the contralateral hemisphere (Blackstad, 1956; Ishizuka et al., 1990). These synapses display an NMDAR-dependent form of synaptic plasticity (Blackstad, 1956; Ishizuka et al., 1990; Debanne et al., 1998). The recurrent fibers of the commissural/associational CA3 projections to CA3 enable a very intense activation of the CA3 pyramidal cells that may play an intrinsic role in long-term memory formation (Marr, 1971; Treves and Rolls, 1994; Nakazawa, 2002; Kesner and Warthen, 2010; Hagena and Manahan-Vaughan, 2012).

This study set about to clarify if long-term synaptic plasticity (>24 h) at the mf-CA3 and commissural/associational-CA3 synapse requires either protein translation or transcription. Our findings support that both LTP and LTD depend on protein transcription but their requirements for protein translation are temporally distinct. This difference is likely to support their functional differentiation with regard to information storage and memory formation.

## Materials and methods

The present study was carried out in accordance with the European Communities Council Directive of September 22nd, 2010 (2010/63/EU) for care of laboratory animals and after approval of the local government ethics committee. All efforts were made to minimize the number of animals used.

### Electrophysiology

**Figure 1:**
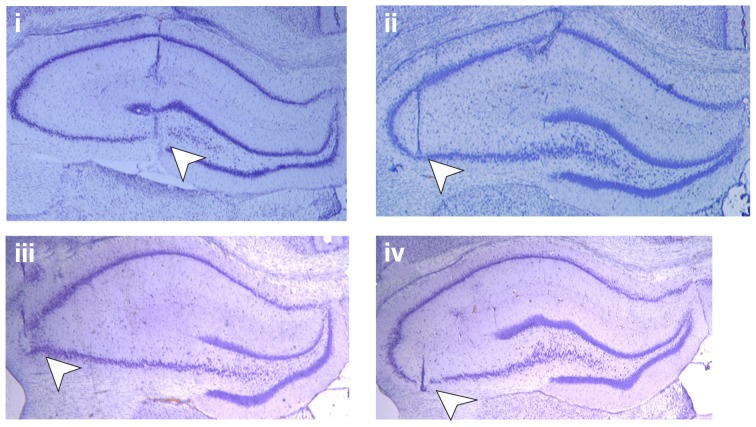
**Position of the electrodes.** Photomicrographs of Nissl-stained hippocampal slices show placement of electrodes (white arrowheads) in mf-CA3 and AC–CA3 preparations: **(i)** shows the position of the stimulation electrode in the mossy fiber pathway and **(ii)** the position of the recording electrode in area CA3 stratum lucidum, whereas picture **(iii)** shows the stimulating electrode in the associational-commissural pathway and **(iv)** the recording electrode in area CA3 stratum radiatum.

Seven- to eight- week old male Wistar rats (Charles River, Germany) were anaesthetized (Pentobarbital, 52 mg/kg, intraperitoneally) and underwent chronic implantation of hippocampal electrodes and a guide cannula, as described previously (Manahan-Vaughan, 1997; Hagena and Manahan-Vaughan, 2011), using coordinates based on the rat brain atlas from Paxinos and Watson (1986). Briefly, for mf-CA3 implantations, the recording electrode was placed above the CA3 pyramidal layer of the dorsal hippocampus, 3.2 mm posterior to bregma and 2.2 mm lateral to midline. The bipolar stimulation electrode was implanted 3.5 mm posterior to bregma and 2.0 mm lateral to midline (Figure 1). For commissural/associational (AC)- CA3 implantations, the recording electrode was placed 3.1 mm posterior tor bregma and 4.2 mm lateral to midline (Figure 1). To verify the correct positions of the electrodes, test pulses were applied to evoke field potentials during the implantation procedure, and postmortem histological analysis was also performed (Bock, 1989; Manahan-Vaughan et al., 1998).

Experiments were commenced 7–10 days after surgery. During all experiments the animals could move freely in the recording chamber (40 × 40 × 50 cm) and had free access to food and water. To allow the animals to acclimatize they were transferred to the experiment room the day before the experiment took place.

Before experiments were begun, animals were stringently assessed to confirm that recordings were obtained from mf—CA3 synapses. The mf synapse is highly sensitive to agonist activation of group II metabotropic glutamate (mGlu) receptors by (2S,2'R,3'R)-2-(2',3'-dicarboxycyclopropyl) glycine (DCG-IV) that selectively inhibits mf but not associational–commissural EPSPs (Kamiya et al., 1996; Yeckel et al., 1999). Animals were excluded from the study when the fEPSP responses evoked in the stratum lucidum failed to show strong sensitivity (i.e., a reduction of test-pulse-evoked responses by 60% or greater) to DCG-IV (20 ng), applied into the lateral cerebral ventricle (i.c.v). To avoid an influence on fEPSPs mediated by DCG-IV on subsequent experiments, it was ensured that potentials had fully recovered before those experiments were performed. In addition, routine studies were conducted to verify that NMDAR-dependent LTP was not evident in these synapses, as described previously (Hagena and Manahan-Vaughan, 2010). Here, 30 min before tetanisation to induce LTP, the NMDAR antagonist, D-(-)-2-amino-5-phosphonopentanoic acid (D-AP5) was applied to examine whether LTP was influenced by the antagonist. Only animals whose LTP was unaffected were included in the current study. For recordings from freely behaving animals, the head stage was connected to an amplifier and stimulator via a flexible cable with a swivel connector. Recordings were analyzed and stored on computer and the EEG was monitored throughout experiments. To evoke fEPSPs a biphasic pulse was given with half-wave duration of 0.2 ms. For recordings, the stimulation intensity was set to produce a fEPSP, which was 40% of the maximal obtainable. The intensity was found on the basis of an input–output curve (maximal stimulation 900 μA). Each recording consisted of an average of five consecutive pulses at 0.025 Hz. To ensure stability of recordings, all animals were first tested in a baseline experiment where test-pulse stimulation was applied over the same time-period as subsequent plasticity experiments. To induce LTD, LFS consisting of 900 pulses at 1 Hz, was given with a stimulus intensity that yielded potentials, which were 70% of the maximal fEPSP observed during the input–output curve analysis. LTP was induced by high-frequency stimulation (HFS) of afferent fibers. This comprised of 4 bursts of 100 pulses at 100 Hz, with a 5 min interburst interval. To ensure a comparability of the evoked potentials, the stimulation was delivered in awake animals within a narrow time window of ±15 min at the same time of the day (ca. 11 a.m.). This protocol was held constant across all animals and experiments.

Animals participating in LTP experiments had a minimum age of 12 weeks, as we observed that application of HFS in younger animals causes epileptiform seizures.

At least regarding effects in the CA1 or dentate gyrus regions, LTP and LTD can be divided into temporally different phases. A distinction can be made between an early phase (early-LTP) that is protein-synthesis independent (Matthies et al., 1990) and a late phase. The early phase has a duration of approximately 2 h (Morrell, 1991) The late phase (late-LTP) relies on protein-synthesis and the induction of immediate early genes (IEGs) (Krug et al., 1984; Frey et al., 1988, 1993, 1996; Otani et al., 1989; Matthies et al., 1990; Kandel, 2001). We thus adopted this terminology in this study.

### Drugs

The reversible protein synthesis inhibitors, (2-[methoxybenzyl]-3,4,pyrrolidinediol 3-acetate) anisomycin (4.8 μg/5 μl; Sigma-Aldrich, Taufkirchen, Germany) and emetine (240 μg/5 μl) were first dissolved in 15 μ l of 1 N HCl solution and then treated with 1 N NaOH to correct for pH. The solutions were subsequently made up to a 50 μ l volume with 0.9% sodium chloride. Actinomycin-D (Sigma-Aldrich) was fully dissolved in 0.9% sodium chloride. A pH of 7.0 was established using 1 N NaOH. The total amount injected was 72 μg/12 μl over a 6 min injection duration. The transcriptional inhibitor 5,6-dichloro-1-beta-D-ribofuranosylbenzamidazole (DRB, 20 nM) was dissolved in 0.9% sodium chloride. The group II mGlu receptor agonist, (2S,2'R,3'R)-2-(2',3'-dicarboxycyclopropyl)glycine (DCG-IV, Tocris Cookson, Bristol, UK) was dissolved in isotonic saline (0.9% NaCl) solution. The amount of DCG-IV that was used (20 ng) was chosen because it has no effect on evoked responses in the dentate gyrus (Klausnitzer and Manahan-Vaughan, 2008). Animals received unilateral injections via the intracerebral ventricle (i.c.v.), specifically via the ipsilateral ventricle by means of the implanted cannula.

The drug dose was dissolved in 5 μl of vehicle and applied over a 5 min period via a Hamilton syringe. To assess if this treatment affected basal synaptic transmission, evoked responses to test-pulse stimulation were assessed over a 24 h period. All injections were carried out 120 min prior to plasticity-evoking stimulation. Drug concentrations were chosen based on a previous study that revealed no effects of actinomycin D, emetine or anisomycin on basal synaptic transmission over a 24 h period (Manahan-Vaughan et al., 2000). The behavioral state of the animal and intrahippocampal EEG were closely monitored to check for adverse reactions to treatment, but none were observed for any of the compounds.

### Data analysis

For each time-point, 5 consecutively evoked responses at 40 s intervals were averaged. The first 30 min of recording (6 time-points) served as baseline, and the results were expressed as the mean percentage ± the standard error of the mean (SEM) of the average baseline value. Baseline values in vehicle-injected animals were recorded for 2 h after injection. In some vehicle experiments, baseline values were obtained for 30 min after injection. Recordings were made every 5 min until 30 min after LFS or HFS and then every 15 min until 4 h had elapsed. On the following day an additional 1 h of recordings was obtained. To examine LTP, HFS of 4 pulses at 100 Hz was applied. To examine LTD, LFS of 900 pulses at 1 Hz was given.

For analysis of difference between groups a two-way analysis of variance (ANOVA) was applied to evaluate the interaction effects between group and time. The level of significance was set at *p* < 0.05.

## Results

### Application of translational inhibitors affect the early and late phases of long-term synaptic plasticity at mossy fiber–CA3 synapses

Treatment with protein-synthesis inhibitors that act on translation, led to an inhibition of the early and late phases of synaptic plasticity in mf-CA3 synapses.

**Figure 2:**
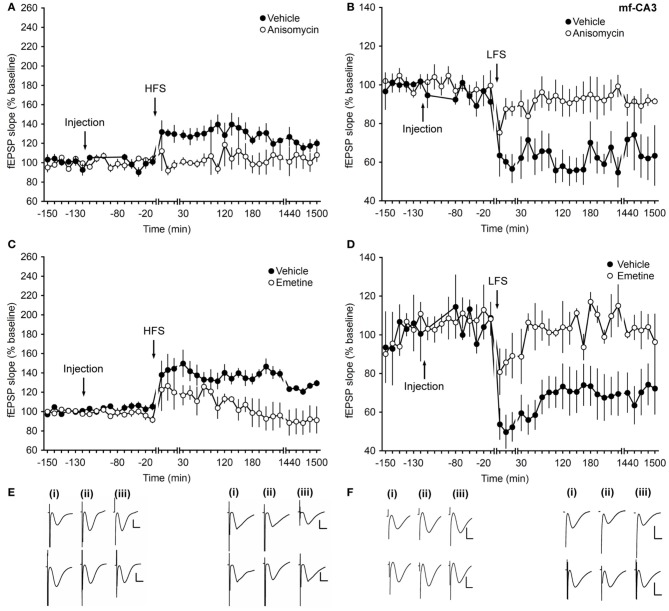
**Application of translational inhibitors affect the early and late phase of long-term synaptic plasticity at mossy fiber–CA3 synapses. (A)** High-frequency stimulation (HFS, 4 trains, 100 Hz) induces LTP that lasts for over 24 h in vehicle-injected animals. Application of the protein-synthesis inhibitor anisomycin (4.8 μg) inhibits the early and late phase of LTP. **(B)** Application of LFS (1 Hz, 900 pulses) induces LTD (>24 h) in vehicle-injected animals. Injection of anisomycin inhibits early- and late LTP. **(C)** Application of HFS elicits LTP (>24 h) in vehicle-injected animals. Injection of emetine (240 μg) inhibits the early and late phase of LTP. **(D)** Injection of emetine leads to an impairment of both the early and late phase of LTD compared to vehicle-injected controls. Line breaks indicate change of time scale. **(E)** Traces in the left panel represent responses recorded during an LTP control experiment (upper traces) and during an experiment where anisomycin was applied prior to HFS (lower traces): analog examples representing pre-HFS **(i)**, 5 min post-HFS **(ii)** and 24 h post-HFS **(iii)** are shown. Vertical scale bar: 2 mV, horizontal scale bar: 8 ms. Analog traces in the right panel are recorded during an experiment in which only LFS was applied (upper traces) and during an anisomycin experiment (lower traces) pre-LFS **(i)**, 5 min post-LFS **(ii)** and 24 h post-LFS **(iii)**. Vertical scale bar: 2 mV, horizontal scale bar: 8 ms. **(F)** Analogs in the left panel depict fEPSPs recorded during a control experiment (upper traces) and during an emetine experiment (lower traces) pre-HFS **(i)**, 5 min post-HFS **(ii)**, and 24 h post-HFS **(iii)**. Vertical scale bar: 2 mV, horizontal scale bar: 8 ms. Analog traces in the right panel represent recordings taken **(i)** pre-LFS, **(ii)** 5 min post-LFS, and **(iii)** 24 h post-LFS in the presence of vehicle (upper traces) or emetine (lower traces). Vertical scale bar: 2 mV, horizontal scale bar: 8 ms.

Robust LTP (>24 h) in vehicle-treated animals was induced with HFS comprising 4 pulses of 100 Hz (Figures 2A,E). Prior treatment with anisomycin resulted in a significant inhibition of the early and late phases of LTP compared to vehicle-treated animals (ANOVA, *F*_(1, 7)_ = 6.04; *p* = 0.04; interaction effect: *F*_(26, 182)_ = 1.558, *p* = 0.049; *n* = 5, Figures 2A,E).

LFS at 1 Hz (900 pulses) elicited long-lasting LTD (>24 h) in vehicle-treated animals (Figures 2B,E). Treatment with anisomycin also resulted in an inhibition of both, the early and late phases of LTD (ANOVA, *F*_(1, 7)_ = 12.95; *p* = 0.008; interaction effect: *F*_(26, 182)_ = 2.9, *p* < 0.0001; *n* = 5, Figures 2B,E).

To confirm these effects, another translation inhibitor, emetine, was used. Application of emetine 2 h before HFS or LFS inhibited the early and late phases of LTP and LTD compared to vehicle-treated controls (ANOVA, *F*_(1, 10)_ = 5.31; *p* = 0.04; interaction effect: *F*_(26, 260)_ = 2.31, *p* < 0.001; *n* = 6, Figures 2C,F and ANOVA, *F*_(1, 5)_ = 10.56; *p* = 0.02; interaction effect: *F*_(26, 130)_ = 1.81, *p* = 0.016; *n* = 4, Figures 2D,F) for HFS and LFS experiments, respectively.

### Inhibitors of transcription affect only the late phase of long-term synaptic plasticity at mossy fiber–CA3 synapses

**Figure 3:**
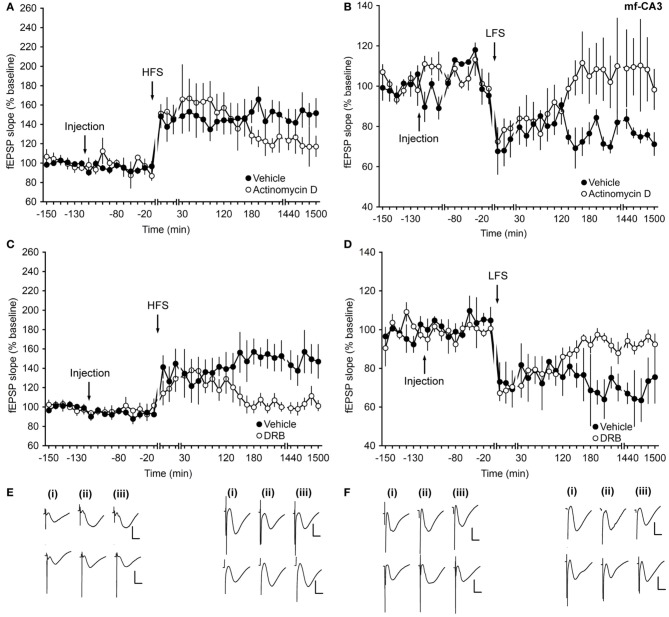
**Inhibitors of transcription affect only the late phase of long-term synaptic plasticity at mossy fiber–CA3 synapses. (A)** In vehicle-injected animals, application of HFS facilitates LTP. When animals are treated with actinomycin D (72 μg) an inhibition of the late phase of LTP occurs. **(B)** LFS elicits LTD in vehicle-injected animals. Application of actinomycin D inhibits the late phase of LTD. **(C)** HFS (4 trains of 100 pulses given at 100 Hz) results in LTP. Injection of the transcription inhibitor DRB (20 nM) inhibits the late phase of LTP. **(D)** In control groups, stimulation with 900 pulses at 1 Hz (LFS) elicits LTD. Injection of DRB inhibits the late phase of LTD. Line breaks indicate change in time scale. **(E)** Analog traces in the left panel were recorded **(i)** pre-HFS, **(ii)** 5 min post-HFS, and **(iii)** 24 h HFS in the presence of vehicle (upper traces) or actinomycin D (lower traces). Vertical scale bar: 2 mV, horizontal scale bar: 8 ms. Analog traces in the right panel represent recordings taken **(i)** pre-LFS, **(ii)** 5 min post-LFS, and **(iii)** 24 h post-LFS in the presence of vehicle (upper traces) or Actinomycin D (lower traces). Vertical scale bar: 2 mV, horizontal scale bar: 8 ms. **(F)** Analog traces in the left panel represent fEPSP responses obtained **(i)** pre-HFS, **(ii)** 5 min post-HFS, and **(iii)** 24 h post-HFS in the presence of vehicle (upper traces) or DRB (lower traces). Vertical scale bar: 2 mV, horizontal scale bar: 8 ms. Analog traces in the right panel represent recordings taken **(i)** pre-LFS, **(ii)** 5 min post-LFS, and **(iii)** 24 h post-LFS in the presence of vehicle (upper traces) or DRB (lower traces). Vertical scale bar: 2 mV, horizontal scale bar: 8 ms.

To elucidate the effect of transcriptional inhibitors on protein-synthesis at mf-CA3 synapses, actinomycin D and DRB were used. Application of actinomycin D 2 h before HFS or LFS resulted in an inhibition of late-LTP (Figures 3A,E) or late-LTD (Figures 3B,E), respectively, compared to controls (ANOVA, *F*_(1, 8)_ = 5.63; *p* = 0.04; interaction effect: *p* = 0.16; *n* = 6 and ANOVA, *F*_(1, 8)_ = 6.7; *p* = 0.03; interaction effect: *p* = 0.06; *n* = 6).

Injection of DRB, similarly resulted in a significant inhibition of the late phase of either LTP (Figures 3C,F) or LTD (Figures 3D,F) after HFS or LFS, respectively, compared to control groups (for HFS experiments: ANOVA, *F*_(1, 8)_ = 5.63; *p* = 0.04; interaction effect: *F*_(15, 120)_ = 2.86, *p* < 0.001; *n* = 5, Figures 3C,F; for LFS experiments: ANOVA, *F*_(1, 6)_ = 8.82; *p* = 0.02; interaction effect: *p* = 0.64; *n* = 4, Figures 3D,F).

### Translation inhibitors prevent the intermediate and late phases of LTP, and solely the late phase of LTD at associational/commissural–CA3 synapses

Since CA3 pyramidal cells do not only form synapses with mossy fibers, but also receive inputs from other CA3 cells via the associational/commissural (AC) fibers (Hjorth-Simonsen, 1973; Swanson et al., 1978; Amaral, 1979; Laurberg, 1979; Ishizuka et al., 1990), we also assessed how protein synthesis inhibitors affect synaptic transmission within these synapses.

**Figure 4:**
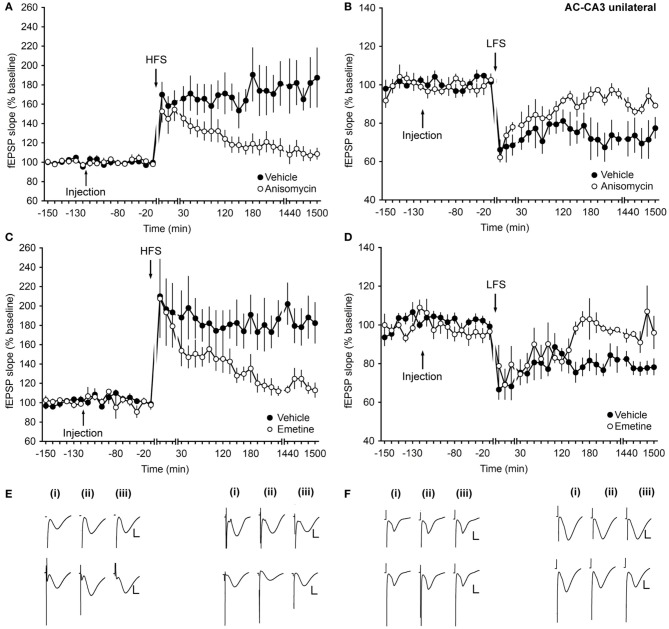
**Translation inhibitors prevent the intermediate and late phases of LTP and solely the late phase of LTD at associational/commissural–CA3 synapses. (A)** Application of HFS results in LTP (>24 h). Treatment with the translational inhibitor anisomycin inhibits the late phase of LTP. **(B)** LFS elicits LTD (>24 h) in control animals. Injection of anisomycin inhibits the late phase of LTD. **(C)** Injection of emetine inhibits the late phase of LTP compared to controls. **(D)** In the presence of emetine, the late phase of LTD is inhibited compared to controls. Line breaks indicate change of time scale. **(E)** Analog traces in the left panel represent recordings taken **(i)** pre-HFS, **(ii)** 5 min post-HFS, and **(iii)** 24 h post-HFS in the presence of vehicle (upper traces) or in anisomycin-injected animals (lower traces). Vertical scale bar: 2 mV, horizontal scale bar: 8 ms. Traces in the right panel show analogs that represent fEPSP responses obtained pre-LFS **(i)**, 5 min post-LFS **(ii)**, and 24 h **(iii)** after LFS in the presence of vehicle (upper traces) or anisomycin (lower traces). Vertical scale bar: 2 mV, horizontal scale bar: 8 ms. **(F)** Analog traces in the left panel represent fEPSP responses recorded pre-HFS **(i)**, 5 min post-HFS **(ii)**, and 24 h after HFS **(iii)** in the presence of vehicle (upper traces) or emetine (lower traces). Vertical scale bar: 2 mV, horizontal scale bar: 8 ms. Analogs in the right panel represent fEPSP responses recorded pre-LFS **(i)**, 5 min post-LFS **(ii)**, and 24 h after LFS **(iii)** in the presence of vehicle (upper traces) or emetine (lower traces). Vertical scale bar: 2 mV, horizontal scale bar: 8 ms.

HFS applied to animals that received vehicle-injection resulted in LTP that lasted for over 24 h. Injection of anisomycin ipsilaterally inhibited LTP starting at ca. 30 min post-HFS (ANOVA, *F*_(1, 13)_ = 11.63; *p* < 0.01; interaction effect: *F*_(23, 299)_ = 2.21, *p* < 0.01; *n* = 9, Figures 4A,E).

Inhibition of late-LTD beginning ca. 2 h after LFS was also evident compared to vehicle-treated controls (ANOVA, *F*_(1, 8)_ = 6.78; *p* = 0.03; interaction effect: *p* = 0.22; *n* = 5, Figures 4B,E).

Emetine treatment confirmed these effects. Here, an inhibition of LTP that began in intermediate phases was apparent (Figures 4C,F). Late–LTD only was affected (Figures 4D,F). (LTP: ANOVA, *F*_(1, 9)_ = 7.86; *p* = 0.02; interaction effect: *F*_(13, 177)_ = 2.16, *p* = 0.02; *n* = 6; LTD: ANOVA, *F*_(1, 5)_ = 9.25; *p* = 0.02; interaction effect: *F*_(14, 70)_ = 1.94, *p* = 0.04; *n* = 5).

### Transcription inhibitors block early and late LTP, and late-LTD only at associational-commissural–CA3 synapses

**Figure 5:**
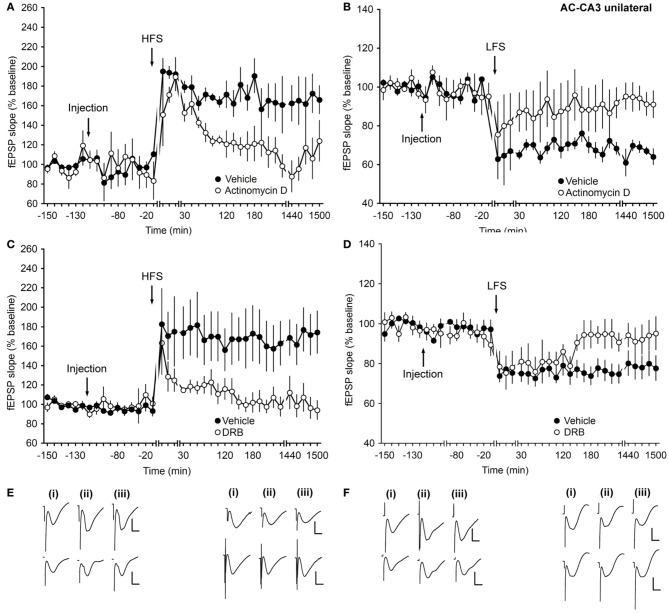
**Transcription inhibitors block early and late LTP, and late-LTD only, at associational/commissural–CA3 synapses. (A)** In control experiments, application of HFS results in LTP (>24 h). Injection of actinomycin D inhibits the early and late phase of LTP. **(B)** Application of LFS results in the expression of LTD (>24 h). In the presence of actinomycin D, early and late phases of LTD are inhibited. **(C)** Injection of DRB inhibits the early and late phase of LTP compared to controls. **(D)** In the presence of DRB, early and late phases of LTD are inhibited compared to controls. Line breaks indicate change in time-scale. **(E)** Traces in the left panel represent fEPSP responses recorded pre-HFS **(i)**, 5 min post-HFS **(ii)**, and 24 h after HFS **(iii)** in the presence of vehicle (upper traces) or actinomycin D (lower traces). Vertical scale bar: 2 mV, horizontal scale bar: 8 ms. Analogs in the right panel represent fEPSP responses recorded pre-LFS **(i)**, 5 min post-LFS **(ii)**, and 24 h after LFS **(iii)** in the presence of vehicle (upper traces) or actinomycin D (lower traces). Vertical scale bar: 2 mV, horizontal scale bar: 8 ms. **(F)** Traces depicted in the left panel represent fEPSP responses recorded pre-HFS **(i)**, 5 min post-HFS **(ii)**, and 24 h after HFS **(iii)** in the presence of vehicle (upper traces) or DRB (lower traces). Vertical scale bar: 2 mV, horizontal scale bar: 8 ms. Analogs in the right panel represent fEPSP responses recorded pre-LFS **(i)**, 5 min post-LFS **(ii)**, and 24 h after LFS **(iii)** in the presence of vehicle (upper traces) or DRB (lower traces). Vertical scale bar: 2 mV, horizontal scale bar: 8 ms.

In control animals, that received only vehicle injection, HFS or LFS led to LTP or LTD, respectively, that lasted for over 24 h (Figures 5A,B). Injection of actinomycin D, ipsilaterally, inhibited the early and late phase of LTP (ANOVA compared to vehicle-injected controls, *F*_(1, 3)_ = 17.20; *p* = 0.02; interaction effect: *p* = 0.43, Figures 5A,E).

Application of actinomycin D, ipsilaterally, inhibited late LTD only (ANOVA compared to vehicle-injected controls, *F*_(1, 6)_=9.34; *p* = 0.02; interaction effect: *p* = 0.07; *n* = 6, Figures 5B,E). Although some effects on intermediate LTD were evident, this was not significant.

Effects were confirmed using DRB, where a significant impairment of the early and late phase of LTP was evident following ipsilateral injection (ANOVA compared to vehicle-injected controls, *F*_(1, 8)_ = 6.01; *p* = 0.03; interaction effect: *F*_(26, 208)_ = 3.97, *p* < 0.001; *n* = 5, Figures 5C,F). LFS in the presence of DRB, resulted, in contrast, in an inhibition only of the late phase of LTD compared to control animals [ANOVA, *F*_(1, 11)_ = 5.12; *p* = 0.04; interaction effect: *p* = 0.81; *n* = 8, Figures 5D,F].

### Application of protein synthesis inhibitors has no effect on basal synaptic transmission in mossy fiber–CA3 synapses

**Figure 6:**
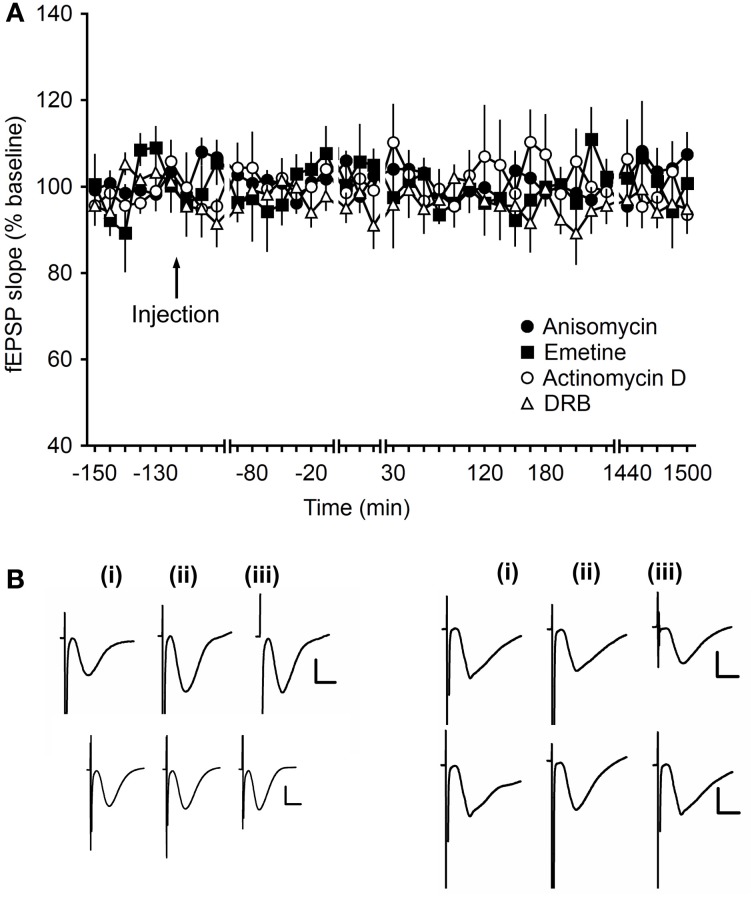
**Application of translational- and transcriptional-Inhibitors do not alter basal synaptic transmission. (A)** After injection of anisomycin (4.8 μg), emetine (240 μg), actinomycin D (72 μg) or DRB (20 nM), fEPSPs recorded during test-pulse stimulation showed stable responses for over 24 h. Line breaks indicate change in time scale. **(B)** Analog traces recorded during test-pulse experiments show fEPSP responses 5 min pre-injection (left traces), 5 min post-injection (middle traces) and 24 h after injection (right traces), in the anisomycin experiment (upper left), in the emetine experiment (lower left), in the actinomycin D experiment (upper right), and in the DRB experiment (lower right). Vertical scale bar: 2 mV, horizontal scale bar: 8 ms.

To assess, whether protein synthesis inhibitors affect basal synaptic transmission in mf-CA3 synapses, in the concentrations used here, the translation inhibitors, anisomycin or emetine, and the transcription inhibitors, actinomycin D or DRB, were applied in an experiment in which animals received only test-pulse stimulation. Here, no effects on the evoked potentials were observed over the 24 monitoring period [ANOVA, *F*_(4, 13)_ = 0.64; *p* = 0.64; *n* = 7, Figures 6A,B].

## Discussion

These data describe for the first time, the protein synthesis dependency of persistent forms of synaptic plasticity at mf-CA3 and AC-CA3 synapses. An interesting picture emerges, that suggests that mf-CA3 and AC-CA3 synapses have distinct requirements for protein translation and transcription. These findings suggest that functionally distinct sources of information to the CA3 region occur, that may relate to its very distinct role in information storage and memory.

Rapid effects of translation inhibitors were seen with regard to both LTP and LTD in mf synapses in our study. This is in line with reports that early mf-CA3 LTP depends on protein translation *in vivo* (Barea-Rodríguez et al., 2000). Protein translation not only occurs in the cell body but also occurs in dendrites after stimulation of afferent fibers (Aakalu et al., 2001; Job and Eberwine, 2001; Smith et al., 2001; Steward and Schuman, 2001; Steward and Worley, 2001). Furthermore, HFS triggers the synthesis of new proteins within minutes (Lynch et al., 1994; Osten et al., 1996; Lanahan and Worley, 1998; Ouyang et al., 1999). MF LTD shares the same mechanisms regarding its induction as LTP (Kobayashi et al., 1996). MF LTD also requires cAMP, which is decreased through activity of presynaptic metabotropic glutamate receptors (mGlus), and requires Rab3A activation (Tzounopoulos et al., 1998). This suggests that LTD, like LTP, might be influenced by the activity of translational inhibitors. A requirement of LTD for rapid protein translation has been described at other hippocampal synapses (Huber et al., 2000). In the light of recent findings on the role of CA3 in spatial learning (Hagena and Manahan-Vaughan, 2011) one can speculate that mossy fibers may be involved in rapid learning of novel information and help in holding that information online for comparison with information stored in AC-CA3 synapses (Hagena and Manahan-Vaughan, 2011). This would in turn explain a need for rapid protein translation.

Inhibition of protein transcription in mf-CA3 synapses prevented the late phases of LTP and LTD and left early phases intact. This is in line with studies that suggest that protein transcription requires several hours (Bliss and Collingridge, 1993; Huang et al., 1994; Nguyen et al., 1994; Nguyen and Kandel, 1996). The stabilization of LTP depends on protein transcription (Calixto et al., 2003). It is not unreasonable to expect that proteins and RNAs necessary for the maintenance of mf LTP are synthesized in the granule cell soma, as high frequency stimulation at other hippocampal synapses upregulates the expression of proteins mediating the release of vesicular neurotransmitters such as synapsin I, synaptotagmin and synaptophysin (Lynch et al., 1994; Hicks et al., 1997). Thus, disruption of a small pool of neurotransmitter-releasing proteins may contribute to an impaired stabilization of LTP. This presynaptic contribution to mf LTP may mediate the maintenance phase, whereas the induction of mf LTP is regulated by postsynaptic mechanisms (Lonart, 2002; Calixto et al., 2003). The importance of the dentate granule cell soma is emphasized by a recent study which shows that action potential-dependent Ca^2+^ influx in granule cell somata as well as fast axonal transport of newly synthesized proteins to mf synapses are required for stable mf LTP (Barnes et al., 2010).

In contrast to results obtained at mf-CA3 synapses, translational inhibitors only affect the late phase of LTP or LTD at AC-CA3 synapses, suggesting that information processing at the AC-synapses is distinct from processing at mf-CA3 synapses. This is not unlikely since it has been speculated that the recurrent network formed by associational fibers is particularly engaged in pattern completion (Guzowski et al., 2004; Lee et al., 2004; Leutgeb et al., 2004; Gold and Kesner, 2005; Rolls and Kesner, 2006), whereas mossy fibers may be important in pattern separation (Bischofberger et al., 2006; Gilbert and Kesner, 2006; Kesner and Hopkins, 2006; Rolls and Kesner, 2006) and the processing of one-trial working memory and the encoding of novel spatial information within a short time-frame (McNaughton and Morris, 1987; Rolls and Kesner, 2006; Kesner, 2007). The postulated role of mf-CA3 synapses in fast processing of rapidly learned information may necessitate rapid protein translation that is not required by AC-synapses.

Strikingly, effects of protein transcription inhibitors on early-LTP and late-LTD were found at AC-CA3 synapses. The fact that LTP and LTD at AC-CA3 synapses are not identically affected by translation and transcription inhibitors, suggests that they play distinct roles in information processing. This possibility is supported by studies of the functional relationship of AC-CA3 synaptic plasticity and spatial memory formation (Hagena and Manahan-Vaughan, 2011). Taken together, a very interesting profile for the protein synthesis-dependency of the CA3 synapses emerges: at mf synapses, dendritic translation assists the early induction of both LTP and LTD akin to effects observed for LTD in the CA1 region (Huber et al., 2000). Dendritic translation does not appear to be required for induction of either LTP or LTD at associational commissural synapses. By contrast, whereas mf synapses do not need transcription for induction of plasticity, at associational-commissural synapses early LTP requires transcription whereas early LTD does not. This latter finding suggests a need for greater stability for AC-LTP compared to LTD at associational-commissural synapses and LTP/LTD at mf synapses.

After HFS, protein-synthesis has a rapid onset with new proteins being built within a time-course of 15–45 min (Lynch et al., 1994; Osten et al., 1996; Lanahan and Worley, 1998; Ouyang et al., 1999). In addition, upregulation of certain RNAs as early as 30 min after plasticity induction has been reported (Sadile et al., 1995; Morimoto et al., 1998; French et al., 2001; Steward and Worley, 2001). Furthermore, IEGs also undergo rapid transcription (Saha et al., 2011), that is implemented for IEG expression changes after LTP induction (Messaoudi et al., 2002) and the IEG Arc is expressed in the CA3 region within seconds of novel context exposure (Pevzner et al., 2012). These findings suggest that very rapid transcriptional effects are feasible and may play a role in synaptic plasticity, particularly in CA3.

The dependency of early AC-LTP on transcription is in line with the possibility that an LTP-specific tag is set by HFS at this synapse. In *in vivo* synapses, one cannot exclude that the synapses are not naïve. A stimulus that is subthreshold for the generation of persistent synaptic plasticity may nonetheless be sufficient to produce a tag in selected synapses. This tag then could capture proteins after a subsequent attempt to induce synaptic plasticity, and support long-lasting modifications of the synapse (Frey and Morris, 1997, 1998; Martin et al., 1997; Casadio et al., 1999; Sajikumar and Frey, 2004; Sajikumar et al., 2007). Protein transcription inhibition would prevent this process, and effects may be detected within minutes of an attempt to induce persistent plasticity. The dependency of early AC-LTP on transcription may be mediated by the setting of such a tag. This could comprise an mRNA or a protein (Sajikumar and Korte, 2011; Li et al., 2012).

We cannot completely exclude however, that the changes of fEPSPs observed after application of transcriptional or translational inhibitors may have been caused by other effects. For example, with regard to anisomycin, it has been shown that, additionally to the well-described action on transcription, it also exhibits neuroprotective effects via regulation of mitogen-activated protein kinases (MAP kinases) and extracellular signal regulated kinases (ERK) (Cano et al., 1994; Hong et al., 2007). Membrane excitability could also have been affected by intrinsic or toxic effects of the compounds (Abbas et al., 2011). However in other studies, where other synapses of the trisynaptic network were scrutinized it was reported that the time course of LTP is unaffected by actinomycin D or anisomycin at the doses used in the current study, suggesting that toxic effects on neurons are negligible under these conditions (Otani et al., 1989; Frey et al., 1996). Furthermore, a specific examination of membrane excitability revealed no effects of actinomycin D (Nikitin and Kozyrev, 2005). However, although emetine was reported to have no effects on intracellularly recorded membrane properties (Stanton and Sarvey, 1984), a more recent study described effects of emetine on basal synaptic transmission in hippocampal slices of young rats *in vitro* (Abbas et al., 2011). We neither saw effects of emetine on basal synaptic transmission in the current study nor in a study of the role of protein synthesis in the CA1 region (Manahan-Vaughan et al., 2000). In both cases stable basal synaptic transmission was followed for 24 h after emetine, anisomycin or actinomycin D treatment. Thus, at the concentration and under the conditions addressed in our study, we assume that no effects on membrane excitability occurred.

The synthesis of new proteins and specifically new mRNA is likely to support synaptic restructuring that is required for the long-term stabilization of synaptic plasticity and new information storage (Bailey et al., 1992; Vanderklish and Edelman, 2002; Sutton and Schuman, 2006). A picture is emerging, whereby it is becoming clear that the protein translation and transcription requirements of the different hippocampal subregions and their respective synapses is very distinct (Otani and Abraham, 1989; Frey et al., 1993; Huang and Kandel, 1994; Nguyen et al., 1994; Manahan-Vaughan et al., 2000; Calixto et al., 2003; Pöschel and Manahan-Vaughan, 2007). In effect, there are few common denominators with regard to the protein synthesis-dependency of LTP and LTD in the different hippocampal subregions. This may relate to the different putative roles of plasticity in these different subregions in information encoding (Kemp and Manahan-Vaughan, 2007; Hagena and Manahan-Vaughan, 2011).

In conclusion, this study describes for the first time a differentiation in the protein translation and protein transcription requirements of persistent LTP and LTD at CA3 synapses *in vivo*. We report that whereas early and late phases of mf-CA3 plasticity are prevented by translation inhibitors, only late phases of mf-CA3 plasticity require transcription. In contrast, only late plasticity of AC-CA3 synapses is affected by protein translation inhibitors, whereas early LTP and late-LTD are affected by transcription inhibitors. These differences are likely to reflect the distinct functional roles of mf-CA3 plasticity and AC-CA3 plasticity in short- and long-term information storage in the hippocampus.

### Conflict of interest statement

The authors declare that the research was conducted in the absence of any commercial or financial relationships that could be construed as a potential conflict of interest.
